# Di-μ-nitrato-bis­(μ-octa­ethyl pyro­phospho­ramide)­bis­[aqua­dinitratocalcium(II)]

**DOI:** 10.1107/S205698902100699X

**Published:** 2021-07-13

**Authors:** Duncan Micallef, Ulrich Baisch

**Affiliations:** a Department of Chemistry, University of Malta, Msida, MSD2080, Malta

**Keywords:** pyro­phospho­ramide, octa­ethyl pyro­phospho­ramide, calcium(II) cation, bridging nitrate anion, Schradan ligand, crystal structure

## Abstract

The title compound, di-μ-nitrato-κ^3^ *O*,*O*′:*O*;*O*:*O*,*O*′-bis­(μ-octa­ethyl pyro­phos­pho­ramide-κ^2^ *O*:*O*′)bis­[aqua­bis­(nitrato-κ^2^ *O*,*O*′)calcium(II)], [Ca_2_(NO_3_)_4_(C_16_H_40_N_4_O_3_P_2_)_2_(H_2_O)_2_] was obtained as a side product during the work up of the synthesis of octa­ethyl pyro­phospho­ramide and represents the first structurally characterized complex of this ligand. The compound crystallizes in the monoclinic space group *P*2_1_/*n* and the asymmetric unit contains one pyro­phospho­ramide mol­ecule and one Ca^2+^ ion coordinated to two nitrate ions and one water mol­ecule. The complex exists as a dimer with a centre of inversion located between two eight-coordinate calcium(II) centres, which are bridged by two nitrate ions and two octa­ethyl pyro­phospho­ramide ligands. Each Ca^2+^ cation is also coordinated to a further nitrate anion, acting as a bidentate ligand, and a water mol­ecule. The complexes stack parallel to the *a* axis and are held in place by a network of inter­molecular O—H⋯O hydrogen bonds also running parallel to *a*.

## Abstract 2

The structure of the first metal complex of octa­ethyl pyro­phospho­ramide is a dimer containing two calcium ions bridged by two nitrate and two octa­ethyl pyro­phospho­ramide ligands. Each calcium ion is further coordinated by a nitrate ion acting as a bidentate ligand and a water mol­ecule and has a coordination number of 8.

## Chemical context   

The structures of octa­ethyl pyro­phospho­ramide, (O((Et_2_N)_2_PO)_2_), and its complexes have not been determined to date. Given the structural similarity of O((Et_2_N)_2_PO)_2_ to the more widely studied Schradan ligand, octa­methyl pyro­phospho­ramide, O((Me_2_N)_2_PO)_2_ (Goehring & Niedenzu, 1956 ▸), it might be expected that the complexes of these two ligands would have related structures. Schradan is known to complex with divalent transition metals and magnesium to form simple chelation complexes of formulae [*M*(O((Me_2_N)_2_PO)_2_)_3_][ClO_4_] (where *M* = Mg^2+^, Cu^2+^ and Co^2+^), in which the metal(II) centre is octa­hedrally coordinated to three pyrophosphate chelate rings (Joesten *et al.*, 1970 ▸) and [Cu(O((Me_2_N)_2_PO)_2_)_2_(ClO_4_)_2_], in which the Cu^II^ atom is coordinated to two pyrophosphate chelate rings and two perchlorate oxygen atoms in an octa­hedral geometry (Hussain *et al.*, 1970 ▸). Schradan has also been reported as a bridging ligand in two dimeric Eu^3+^ complexes (Chan *et al.*, 2020 ▸). Here we report what we believe to be the first example of a metal-coordinated octa­ethyl pyro­phospho­ramide complex, which is dimeric and has the formula [Ca(O((Et_2_N)_2_PO)_2_(NO_3_)_2_(H_2_O)]_2_.

## Structural commentary   

The asymmetric unit contains one pyro­phospho­ramide mol­ecule together with one Ca^2+^ ion coordinated to two nitrates and one water mol­ecule. None of the atoms lie on special positions. The content of one asymmetric unit makes up one half of the actual dimeric calcium complex, which has a centre of inversion midway between the two calcium atoms, bringing *Z* to 2. 
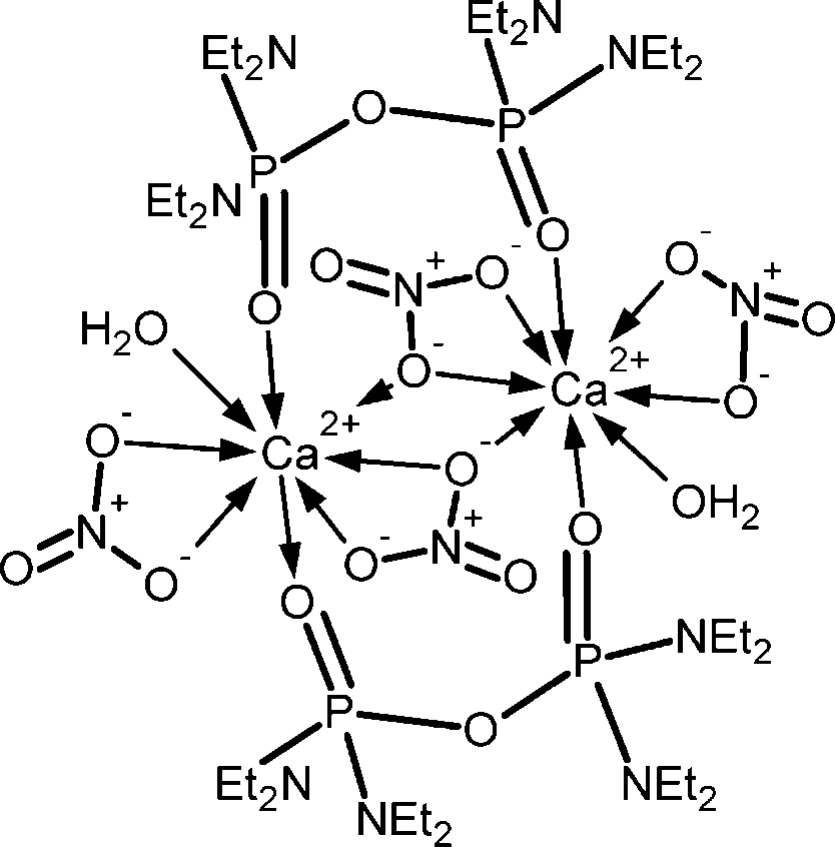


**Figure 1:**
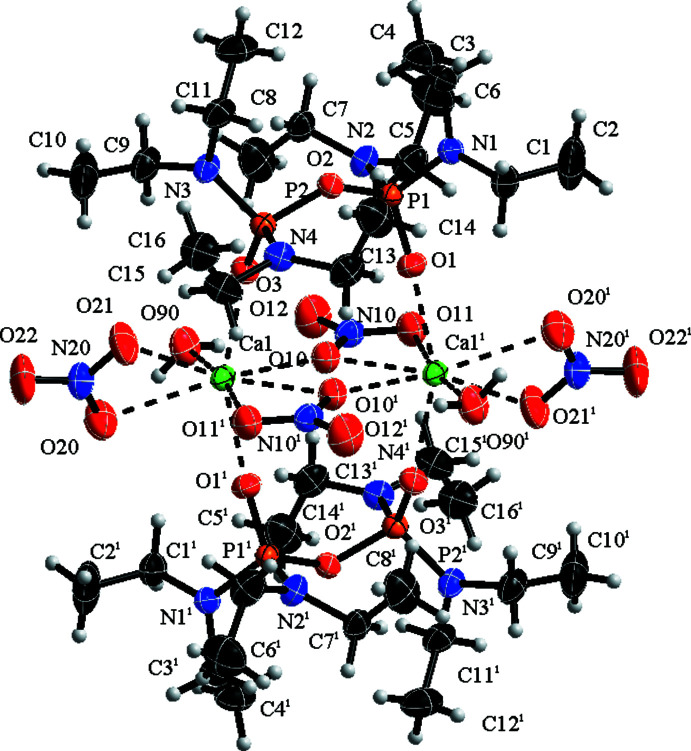
Mol­ecular structure of (I). Displacement ellipsoids of all non-hydrogen atoms are drawn at the 70% probability level. Dashed bonds highlight the eight-coordination around the Ca^2+^ cations. [Symmetry code: (i) −*x* + 1, −*y* + 1, −*z* + 1].

In the title complex (Fig. 1 ▸), the di-N-substituted pyro­phospho­ramide mol­ecule acts as a bridging ligand, rather than a bidentate chelating ligand, unlike in the previously characterized transition-metal and alkaline-earth metal complexes of the Schradan ligand, O((Me_2_N)_2_PO)_2_ (Joesten *et al.*, 1970 ▸; Hussain *et al.*, 1970 ▸).

The coordination number of the Ca^2+^ cation in the title compound is eight, which is typical for Ca^2+^ complexes. There are two Ca—O(P=O) bond lengths per O((Et_2_N)_2_PO)_2_ ligand, Ca1—O1 and Ca1—O3^i^ with 2.3054 (13) and 2.3324 (13) Å, respectively; (Table 1 ▸), both of which are rather longer than the average lengths for analogous bonds found in simple phospho­ramide complexes of Ca^2+^ (see *Database Survey* below). The corresponding P=O bond lengths in the O((Et_2_N)_2_PO)_2_ ligand, P1—O1 and P2—O3, are 1.4752 (13) and 1.4722 (13) Å, respectively (Table 1 ▸) and are comparable to values reported in other complexes where the ligands are also coordinated *via* the P=O moiety.

## Supra­molecular features   

**Figure 2:**
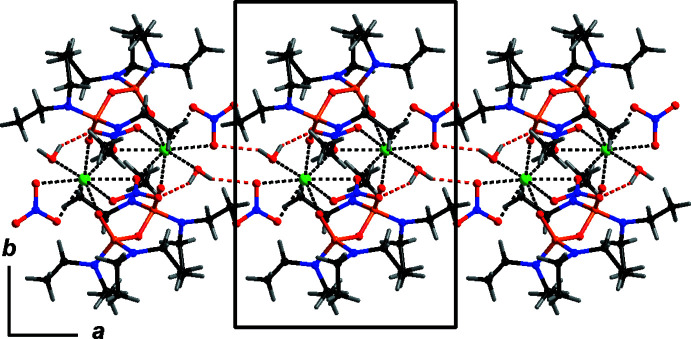
Inter­molecular and intra­molecular N—O⋯H—O hydrogen bonding (red broken-off bonds) that create the packing chains along the *a*-axis direction, viewed along the *c* axis. Dashed black bonds highlight the eight-coordination around the Ca^2+^ cations.

The complexes pack to form chains running along the *a*-axis direction, where neighbouring complexes are bound by inter­molecular hydrogen bonding of the type H—O⋯O—N, as shown in Fig. 2 ▸, involving the aqua ligand and the non-bridging nitrate anion, namely O90—H90*A*⋯O20 (Table 2 ▸). The aqua ligand also forms a hydrogen-bonding motif with the bridging nitrate anion, namely O90—H90*B*⋯O12 (Table 2 ▸).

## Database survey   

All searches were carried out using the Cambridge Structural Database (CSD Version 5.41, last update May 2020; Groom *et al.*, 2016 ▸). A search for the structure of octa­ethyl pyro­phospho­ramide and its complexes returned no hits. A search for the structure of octa­methyl pyro­phospho­ramide (Schradan) and its complexes returned six hits in which the ligand was found to chelate with the metal cations. Of these, four were octa­hedral metal complexes of this ligand with magnesium: [Mg(O((Me_2_N)_2_PO)_2_)_3_][ClO_4_] (MEPOMG; Joesten *et al.*, 1970 ▸), cobalt: [Co(O((Me_2_N)_2_PO)_2_)_3_][ClO_4_] (MEPOCO; Joesten *et al.*, 1970 ▸) and copper: [Cu(O((Me_2_N)_2_PO)_2_)_3_][ClO_4_] (MPAMCU10; Joesten *et al.*, 1970 ▸) and [Cu(O((Me_2_N)_2_PO)_2_)_2_(ClO_4_)_2_] (OMPOCU; Hussain *et al.*, 1970 ▸), and two were eight-coordinate metal complexes with actinides: [U(O((Me_2_N)_2_PO)_2_)_2_(NCS)_4_] (BOXXUH) and [Th(O((Me_2_N)_2_PO)_2_)_2_Cl_4_] (BOXYAO) (Kepert *et al.*, 1983 ▸). Two further hits were found in which the Schradan ligand formed a bridge between two seven-coordinate Eu^3+^ ions in the complexes [(dmp-*O*,*O*′)_3_Eu((O((Me_2_N)_2_PO)_2_)Eu(*O*,*O*′-dmp)_3_] (dmp = [HC(C(^t^Bu)·CO)_2_]^−^) (KUXTOP, KUXVIL; Chan *et al.*, 2020 ▸).

A similar search for other di-N-substituted pyro­phospho­ramide complexes returned no hits, whilst a search for mono-N-substituted pyro­phospho­ramide complexes returned one hit, namely the octa­hedral complex, [Mn(O((*t*BuNH)_2_PO)_2_)_2_(DMF)_2_][Cl]_2_·2H_2_O (PEWRAM), in which the pyro­phospho­ramide ligand was found to chelate to a mangan­ese(II) cation (Tarahhomi *et al.*, 2013 ▸).

Although no pyro­phospho­ramide complexes of calcium were found, a search for di-λ^5^σ^4^-phospho­rane species containing the fragment O=P—*X*—P=O—Ca yielded 17 hits. The complex tris­(μ_2_-tetra­phenyl­imidophosphinato-*O*,*O*,*O*′)aqua­(tetra­phenyl­imidophosphinato-*O*,*O*′)dicalcium (VAYQUI; Morales-Juárez *et al.*, 2005 ▸) was the only species found to contain the O=P—*X*—P=O—Ca fragment bridging two Ca^2+^ cations that did not form part of a cluster or polymer. However in this case, both calcium centres have a coordination number of six, with distorted octa­hedral geometries, and bridging is achieved *via* one μ-oxygen atom per [N(Ph_2_PO)_2_]^−^ ligand. This is, however, unlike the bridging behaviour observed in the title complex.

## Synthesis and crystallization   

The title compound was obtained as a minor component on purification of octa­ethyl pyro­phospho­ramide through column chromatography. The synthesis of octa­ethyl pyro­phospho­ramide was undertaken using standard Schlenk line techniques. All solvents were dried over 4 Å mol­ecular sieves. An excess amount of di­ethyl­amine (used as purchased), namely 7.6 ml (0.073 mol), was dissolved in 10 ml of chloro­form. The solution was cooled to 195 K and 1 ml (0.007 mol) of pyro­phosphoryl chloride (purified by short-path distillation) was added dropwise using a glass syringe with constant stirring. After the addition was complete, the cooling bath was removed and the mixture allowed to react at room temperature overnight with continuous stirring. Approximately 15 ml of *n*-pentane was then added to yield a deep-red-coloured suspension and this was left overnight to allow precipitation. The suspension was filtered using a series of cannula filtrations to remove the di­ethyl­ammonium chloride by-product. Volatile products were removed under vacuum at 323 K. This yielded the crude octa­ethyl pyro­phospho­ramide as a viscous red liquid. This was subsequently purified by column chromatography using a dilute nitric-acid-activated Kieselgel 60 as the stationary phase and di­chloro­methane/aceto­nitrile as eluents. Octa­ethyl pyro­phospho­ramide was collected in aceto­nitrile as a dark-pink viscous liquid after removal of volatiles under vacuum at room temperature.

On storage of the liquid octa­ethyl pyro­phospho­ramide product over a number of weeks, single crystals of the title compound formed serendipitously. Introduction of Ca^2+^ and NO_3_^−^ ions most likely arose from either the use of dilute nitric acid in the activation process of the silica gel used for column chromatography or from impurities present in the mol­ecular sieve. Both the Kieselgel 60 and the mol­ecular sieve were not used as received from the supplier, but were reused following washing/cleaning partly with nitric acid. The Ca^2+^ ions may have been introduced from previous use and remained inside the column or drying material.

## Refinement   

Crystal data, data collection and structure refinement details are summarized in Table 3 ▸. H atoms were positioned geometrically (O—H = 0.87, C—H = 0.98–0.99 Å) and refined as riding with *U*_iso_(H) = 1.2*U*_eq_(C) or 1.5*U*_eq_(O, C-meth­yl).

## Supplementary Material

Crystal structure: contains datablock(s) I. DOI: 10.1107/S205698902100699X/cq2040sup1.cif

Structure factors: contains datablock(s) I. DOI: 10.1107/S205698902100699X/cq2040Isup2.hkl

CCDC reference: 2094905

Additional supporting information:  crystallographic information; 3D view; checkCIF report

## Figures and Tables

**Table 1:** Selected bond lengths (Å)

Ca1—O3^i^	2.3324 (13)	P2—O3	1.4722 (13)
Ca1—O1	2.3054 (13)	P1—O1	1.4752 (13)

Symmetry code: (i) -x+1, -y+1, -z+1.

**Table 2:** Hydrogen-bond geometry (Å, °)

*D*—H⋯*A*	*D*—H	H⋯*A*	*D*⋯*A*	*D*—H⋯*A*
O90—H90*A*⋯O20^ii^	0.87	2.21	2.915 (2)	138
O90—H90*B*⋯O10	0.87	2.58	2.9568 (19)	108
O90—H90*B*⋯O12	0.87	2.11	2.913 (2)	153
C1—H1*B*⋯O1	0.99	2.40	2.929 (2)	113
C7—H7*A*⋯O2	0.99	2.39	2.920 (2)	113
C9—H9*B*⋯O3	0.99	2.45	2.967 (2)	112

Symmetry code: (ii) -x+2, -y+1, -z+1.

**Table 3:** Experimental details

Crystal data	
Chemical formula	[Ca_2_(NO_3_)_4_(C_16_H_40_N_4_O_3_P_2_)_2_(H_2_O)_2_]
*M*_r_	1161.15
Crystal system, space group	Monoclinic, *P*2_1_/*n*
Temperature (K)	150
*a*, *b*, *c* (Å)	10.6249 (7), 15.5774 (12), 17.0925 (10)
β (°)	96.707 (5)
*V* (Å^3^)	2809.6 (3)
*Z*	2
Radiation type	Cu *K*α
μ (mm^−1^)	3.50
Crystal size (mm)	0.21 × 0.07 × 0.06

Data collection	
Diffractometer	Stoe Stadivari
Absorption correction	Integration (*X-RED32*; Stoe & Cie, 2020 ▸),
*T*_min_, *T*_max_	0.587, 0.827
No. of measured, independent and observed [*I* > 2σ(*I*)] reflections	4816, 4816, 4280
*R*_int_	0.104
(sin θ/λ)_max_ (Å^−1^)	0.591

Refinement	
*R*[*F* ^2^ > 2σ(*F* ^2^)], *wR*(*F* ^2^), *S*	0.039, 0.114, 1.06
No. of reflections	4816
No. of parameters	325
H-atom treatment	H-atom parameters constrained
Δρ_max_, Δρ_min_ (e Å^−3^)	0.57, −0.51

Computer programs: *X-AREA Recipe* and *Integrate* and *X-RED* (Stoe & Cie, 2020 ▸), *SHELXT* (Sheldrick, 2015*a* ▸), *SHELXL* (Sheldrick, 2015*b* ▸) and *OLEX2* (Dolomanov *et al.*, 2009 ▸).

## supplementary crystallographic information

### Crystal data

**Table:**

[Ca_2_(NO_3_)_4_(C_16_H_40_N_4_O_3_P_2_)_2_(H_2_O)_2_]	*F*(000) = 1240
*M**_r_* = 1161.15	*D*_x_ = 1.373 Mg m^−^^3^
Monoclinic, *P*2_1_/*n*	Cu *K*α radiation, λ = 1.54186 Å
*a* = 10.6249 (7) Å	Cell parameters from 18894 reflections
*b* = 15.5774 (12) Å	θ = 2.2–50.0°
*c* = 17.0925 (10) Å	µ = 3.50 mm^−^^1^
β = 96.707 (5)°	*T* = 150 K
*V* = 2809.6 (3) Å^3^	Block, clear colourless
*Z* = 2	0.21 × 0.07 × 0.06 mm

### Data collection

**Table:**

Stoe Stadivari diffractometer	4816 independent reflections
Radiation source: Genix-Cu	4280 reflections with *I* > 2σ(*I*)
Graded multilayer mirror monochromator	*R*_int_ = 0.104
Detector resolution: 5.81 pixels mm^-1^	θ_max_ = 65.7°, θ_min_ = 3.9°
rotation method, ω scans	*h* = −12→9
Absorption correction: integration (X-RED32; Stoe & Cie, 2020),	*k* = −18→17
*T*_min_ = 0.587, *T*_max_ = 0.827	*l* = −20→15
4816 measured reflections	

### Refinement

**Table:**

Refinement on *F*^2^	Primary atom site location: dual
Least-squares matrix: full	Hydrogen site location: mixed
*R*[*F*^2^ > 2σ(*F*^2^)] = 0.039	H-atom parameters constrained
*wR*(*F*^2^) = 0.114	*w* = 1/[σ^2^(*F*_o_^2^) + (0.0894*P*)^2^] where *P* = (*F*_o_^2^ + 2*F*_c_^2^)/3
*S* = 1.06	(Δ/σ)_max_ = 0.001
4816 reflections	Δρ_max_ = 0.57 e Å^−^^3^
325 parameters	Δρ_min_ = −0.51 e Å^−^^3^
0 restraints	

### Special details

**Table:**

Geometry. All esds (except the esd in the dihedral angle between two l.s. planes) are estimated using the full covariance matrix. The cell esds are taken into account individually in the estimation of esds in distances, angles and torsion angles; correlations between esds in cell parameters are only used when they are defined by crystal symmetry. An approximate (isotropic) treatment of cell esds is used for estimating esds involving l.s. planes.

### Fractional atomic coordinates and isotropic or equivalent isotropic displacement parameters (Å^2^)

**Table:**

	*x*	*y*	*z*	*U*_iso_*/*U*_eq_	
Ca1	0.68362 (3)	0.54553 (2)	0.47940 (2)	0.01850 (13)	
P2	0.45347 (4)	0.26727 (3)	0.43162 (3)	0.01774 (14)	
P1	0.62937 (4)	0.35998 (3)	0.33838 (3)	0.01807 (14)	
O3	0.38108 (12)	0.34680 (8)	0.43931 (8)	0.0219 (3)	
O10	0.55306 (12)	0.45527 (8)	0.56079 (8)	0.0239 (3)	
O2	0.56085 (12)	0.27924 (8)	0.37352 (7)	0.0197 (3)	
O1	0.66242 (13)	0.42666 (9)	0.39857 (8)	0.0248 (3)	
O90	0.83004 (13)	0.48374 (10)	0.57766 (9)	0.0330 (3)	
H90A	0.888725	0.456432	0.556513	0.049*	
H90B	0.793535	0.443861	0.602403	0.049*	
O11	0.44513 (13)	0.38712 (9)	0.64024 (8)	0.0294 (3)	
O12	0.64962 (14)	0.39723 (11)	0.66660 (9)	0.0413 (4)	
O20	0.90314 (14)	0.55869 (10)	0.42435 (10)	0.0376 (4)	
O21	0.80964 (15)	0.67526 (10)	0.45115 (11)	0.0437 (4)	
N10	0.55055 (15)	0.41293 (10)	0.62398 (9)	0.0237 (4)	
N2	0.53139 (15)	0.38796 (10)	0.26229 (10)	0.0237 (4)	
N3	0.36202 (14)	0.19088 (10)	0.39291 (10)	0.0227 (4)	
O22	0.99372 (15)	0.68139 (11)	0.40806 (12)	0.0477 (5)	
N1	0.75741 (15)	0.32132 (11)	0.30813 (10)	0.0242 (4)	
N20	0.90434 (15)	0.63966 (11)	0.42762 (11)	0.0293 (4)	
N4	0.53719 (15)	0.22829 (10)	0.50962 (10)	0.0237 (4)	
C11	0.4177 (2)	0.10937 (12)	0.37085 (12)	0.0273 (4)	
H11A	0.367488	0.061459	0.389243	0.033*	
H11B	0.504756	0.104824	0.398423	0.033*	
C3	0.7476 (2)	0.25653 (14)	0.24476 (12)	0.0279 (4)	
H3A	0.812391	0.269205	0.209197	0.033*	
H3B	0.663391	0.261844	0.213587	0.033*	
C1	0.88159 (19)	0.33081 (15)	0.35595 (13)	0.0309 (5)	
H1A	0.911268	0.273725	0.375788	0.037*	
H1B	0.871733	0.367625	0.402055	0.037*	
C7	0.41259 (18)	0.34500 (13)	0.23158 (12)	0.0260 (4)	
H7A	0.403116	0.292083	0.262431	0.031*	
H7B	0.417409	0.327939	0.176221	0.031*	
C13	0.6738 (2)	0.24323 (14)	0.53053 (13)	0.0311 (5)	
H13A	0.690616	0.252103	0.588158	0.037*	
H13B	0.697767	0.296606	0.504532	0.037*	
C5	0.5696 (2)	0.46275 (14)	0.21772 (13)	0.0333 (5)	
H5A	0.647887	0.487429	0.246018	0.040*	
H5B	0.502460	0.506988	0.216419	0.040*	
C9	0.22450 (18)	0.20147 (14)	0.37095 (14)	0.0309 (5)	
H9A	0.203236	0.186627	0.314643	0.037*	
H9B	0.201501	0.262377	0.377774	0.037*	
C15	0.4636 (2)	0.19640 (13)	0.57244 (13)	0.0305 (5)	
H15A	0.373954	0.213873	0.559605	0.037*	
H15B	0.496278	0.224166	0.622840	0.037*	
C12	0.4233 (2)	0.09937 (15)	0.28312 (14)	0.0391 (5)	
H12A	0.337590	0.103820	0.255194	0.059*	
H12B	0.459172	0.043147	0.272656	0.059*	
H12C	0.476725	0.144696	0.264789	0.059*	
C4	0.7648 (2)	0.16493 (14)	0.27330 (14)	0.0370 (5)	
H4A	0.748221	0.125643	0.228494	0.056*	
H4B	0.705410	0.152915	0.311699	0.056*	
H4C	0.851828	0.156853	0.298193	0.056*	
C16	0.4690 (2)	0.10042 (14)	0.58328 (14)	0.0381 (5)	
H16A	0.430404	0.072327	0.535044	0.057*	
H16B	0.422535	0.084300	0.627335	0.057*	
H16C	0.557539	0.082205	0.594521	0.057*	
C8	0.2968 (2)	0.40106 (18)	0.23508 (16)	0.0452 (6)	
H8A	0.304985	0.453303	0.204137	0.068*	
H8B	0.289688	0.416577	0.289955	0.068*	
H8C	0.220780	0.369542	0.213405	0.068*	
C14	0.7567 (2)	0.17060 (17)	0.50733 (15)	0.0421 (6)	
H14A	0.742998	0.162586	0.450102	0.063*	
H14B	0.734829	0.117608	0.533536	0.063*	
H14C	0.845906	0.184628	0.523349	0.063*	
C10	0.1470 (2)	0.14537 (17)	0.42031 (18)	0.0491 (7)	
H10A	0.056894	0.150505	0.400662	0.074*	
H10B	0.160571	0.164080	0.475393	0.074*	
H10C	0.173586	0.085408	0.416665	0.074*	
C2	0.9788 (2)	0.3693 (2)	0.31029 (19)	0.0584 (8)	
H2A	0.996162	0.329816	0.268210	0.088*	
H2B	1.056983	0.379490	0.345473	0.088*	
H2C	0.947115	0.423835	0.287175	0.088*	
C6	0.5930 (3)	0.44255 (18)	0.13388 (15)	0.0484 (7)	
H6A	0.514821	0.420815	0.104470	0.073*	
H6B	0.659625	0.398951	0.134411	0.073*	
H6C	0.619838	0.494832	0.108560	0.073*	

### Atomic displacement parameters (Å^2^)

**Table:**

	*U*^11^	*U*^22^	*U*^33^	*U*^12^	*U*^13^	*U*^23^
Ca1	0.0150 (2)	0.0179 (2)	0.0220 (2)	0.00120 (13)	−0.00005 (15)	−0.00325 (14)
P2	0.0174 (2)	0.0155 (2)	0.0201 (3)	0.00135 (17)	0.00129 (18)	−0.00050 (17)
P1	0.0152 (2)	0.0185 (2)	0.0203 (3)	−0.00014 (17)	0.00136 (18)	−0.00302 (17)
O3	0.0221 (7)	0.0179 (6)	0.0259 (7)	0.0036 (5)	0.0036 (5)	−0.0010 (5)
O10	0.0207 (7)	0.0263 (7)	0.0240 (7)	0.0012 (5)	−0.0004 (5)	0.0040 (6)
O2	0.0189 (6)	0.0183 (6)	0.0223 (7)	0.0015 (5)	0.0039 (5)	−0.0021 (5)
O1	0.0248 (7)	0.0230 (7)	0.0265 (7)	−0.0019 (5)	0.0019 (6)	−0.0075 (6)
O90	0.0198 (7)	0.0419 (9)	0.0362 (8)	0.0058 (6)	−0.0012 (6)	0.0058 (7)
O11	0.0276 (8)	0.0311 (8)	0.0299 (8)	−0.0009 (6)	0.0047 (6)	0.0036 (6)
O12	0.0275 (8)	0.0603 (11)	0.0330 (8)	0.0050 (7)	−0.0095 (7)	0.0104 (8)
O20	0.0310 (8)	0.0274 (8)	0.0563 (10)	0.0044 (6)	0.0126 (7)	0.0017 (7)
O21	0.0286 (8)	0.0315 (8)	0.0752 (12)	0.0014 (7)	0.0237 (8)	−0.0035 (8)
N10	0.0212 (8)	0.0255 (8)	0.0233 (8)	0.0021 (7)	−0.0017 (7)	−0.0021 (7)
N2	0.0221 (8)	0.0219 (8)	0.0262 (9)	−0.0028 (6)	−0.0008 (7)	0.0029 (7)
N3	0.0184 (8)	0.0170 (8)	0.0322 (9)	0.0005 (6)	0.0009 (7)	−0.0020 (6)
O22	0.0269 (8)	0.0472 (10)	0.0717 (13)	−0.0090 (7)	0.0167 (8)	0.0101 (9)
N1	0.0166 (8)	0.0296 (9)	0.0262 (8)	0.0005 (6)	0.0023 (6)	−0.0074 (7)
N20	0.0186 (8)	0.0334 (10)	0.0356 (10)	−0.0012 (7)	0.0021 (7)	0.0036 (8)
N4	0.0250 (9)	0.0236 (8)	0.0220 (8)	0.0029 (7)	0.0008 (7)	0.0028 (6)
C11	0.0276 (10)	0.0171 (9)	0.0359 (11)	0.0023 (8)	−0.0017 (9)	−0.0040 (8)
C3	0.0256 (10)	0.0331 (11)	0.0258 (10)	0.0010 (8)	0.0069 (8)	−0.0087 (8)
C1	0.0182 (10)	0.0375 (12)	0.0362 (11)	−0.0003 (8)	0.0003 (8)	−0.0053 (9)
C7	0.0193 (9)	0.0318 (11)	0.0255 (10)	−0.0024 (8)	−0.0032 (8)	−0.0005 (8)
C13	0.0286 (11)	0.0357 (12)	0.0261 (10)	0.0011 (9)	−0.0085 (8)	0.0020 (9)
C5	0.0388 (12)	0.0275 (11)	0.0322 (12)	−0.0067 (9)	−0.0019 (9)	0.0066 (9)
C9	0.0207 (10)	0.0276 (11)	0.0428 (12)	0.0002 (8)	−0.0034 (9)	−0.0020 (9)
C15	0.0394 (12)	0.0272 (11)	0.0257 (10)	0.0070 (9)	0.0071 (9)	0.0058 (8)
C12	0.0423 (13)	0.0349 (12)	0.0383 (13)	0.0086 (10)	−0.0034 (10)	−0.0133 (10)
C4	0.0397 (13)	0.0318 (12)	0.0391 (13)	0.0106 (10)	0.0025 (10)	−0.0097 (10)
C16	0.0471 (14)	0.0286 (12)	0.0392 (13)	0.0002 (10)	0.0071 (10)	0.0075 (10)
C8	0.0253 (11)	0.0602 (17)	0.0486 (15)	0.0117 (11)	−0.0026 (10)	0.0055 (12)
C14	0.0294 (12)	0.0568 (15)	0.0392 (13)	0.0101 (11)	0.0007 (10)	0.0013 (11)
C10	0.0263 (12)	0.0461 (15)	0.076 (2)	−0.0087 (11)	0.0096 (12)	0.0008 (13)
C2	0.0279 (13)	0.081 (2)	0.0661 (19)	−0.0202 (14)	0.0037 (12)	0.0075 (16)
C6	0.0620 (17)	0.0476 (15)	0.0385 (14)	−0.0005 (13)	0.0184 (12)	0.0105 (11)

### Geometric parameters (Å, º)

**Table:**

Ca1—Ca1^i^	4.2866 (7)	C1—H1B	0.9900
Ca1—O3^i^	2.3324 (13)	C1—C2	1.491 (3)
Ca1—O10	2.5075 (13)	C7—H7A	0.9900
Ca1—O10^i^	2.5283 (14)	C7—H7B	0.9900
Ca1—O1	2.3054 (13)	C7—C8	1.516 (3)
Ca1—O90	2.3574 (14)	C13—H13A	0.9900
Ca1—O11^i^	2.5495 (15)	C13—H13B	0.9900
Ca1—O20	2.6230 (15)	C13—C14	1.515 (3)
Ca1—O21	2.5022 (16)	C5—H5A	0.9900
Ca1—N10^i^	2.9503 (16)	C5—H5B	0.9900
Ca1—N20	2.9863 (17)	C5—C6	1.516 (3)
P2—O3	1.4722 (13)	C9—H9A	0.9900
P2—O2	1.6084 (13)	C9—H9B	0.9900
P2—N3	1.6272 (16)	C9—C10	1.522 (3)
P2—N4	1.6312 (16)	C15—H15A	0.9900
P1—O2	1.6046 (13)	C15—H15B	0.9900
P1—O1	1.4752 (13)	C15—C16	1.507 (3)
P1—N2	1.6276 (16)	C12—H12A	0.9800
P1—N1	1.6264 (16)	C12—H12B	0.9800
O10—N10	1.268 (2)	C12—H12C	0.9800
O90—H90A	0.8678	C4—H4A	0.9800
O90—H90B	0.8681	C4—H4B	0.9800
O11—N10	1.251 (2)	C4—H4C	0.9800
O12—N10	1.233 (2)	C16—H16A	0.9800
O20—N20	1.263 (2)	C16—H16B	0.9800
O21—N20	1.255 (2)	C16—H16C	0.9800
N2—C7	1.470 (2)	C8—H8A	0.9800
N2—C5	1.475 (2)	C8—H8B	0.9800
N3—C11	1.469 (2)	C8—H8C	0.9800
N3—C9	1.475 (2)	C14—H14A	0.9800
O22—N20	1.229 (2)	C14—H14B	0.9800
N1—C3	1.475 (3)	C14—H14C	0.9800
N1—C1	1.476 (2)	C10—H10A	0.9800
N4—C13	1.472 (3)	C10—H10B	0.9800
N4—C15	1.486 (3)	C10—H10C	0.9800
C11—H11A	0.9900	C2—H2A	0.9800
C11—H11B	0.9900	C2—H2B	0.9800
C11—C12	1.516 (3)	C2—H2C	0.9800
C3—H3A	0.9900	C6—H6A	0.9800
C3—H3B	0.9900	C6—H6B	0.9800
C3—C4	1.512 (3)	C6—H6C	0.9800
C1—H1A	0.9900		
			
O3^i^—Ca1—O10^i^	79.18 (5)	C1—N1—P1	120.90 (13)
O3^i^—Ca1—O10	81.49 (5)	O20—N20—Ca1	61.21 (10)
O3^i^—Ca1—O90	94.88 (5)	O21—N20—Ca1	55.63 (10)
O3^i^—Ca1—H90A	110.2	O21—N20—O20	116.82 (17)
O3^i^—Ca1—H90B	94.9	O22—N20—Ca1	177.28 (15)
O3^i^—Ca1—O11^i^	90.80 (5)	O22—N20—O20	121.36 (18)
O3^i^—Ca1—O20	119.59 (5)	O22—N20—O21	121.82 (18)
O3^i^—Ca1—O21	74.68 (5)	C13—N4—P2	124.58 (14)
O3^i^—Ca1—N10^i^	84.98 (5)	C13—N4—C15	117.69 (17)
O3^i^—Ca1—N20	96.88 (5)	C15—N4—P2	115.62 (14)
O10—Ca1—O10^i^	63.31 (5)	N3—C11—H11A	108.8
O10^i^—Ca1—H90A	145.6	N3—C11—H11B	108.8
O10—Ca1—H90A	84.9	N3—C11—C12	113.97 (17)
O10^i^—Ca1—H90B	121.3	H11A—C11—H11B	107.7
O10—Ca1—H90B	58.1	C12—C11—H11A	108.8
O10—Ca1—O11^i^	113.32 (5)	C12—C11—H11B	108.8
O10^i^—Ca1—O11^i^	50.25 (4)	N1—C3—H3A	108.7
O10—Ca1—O20	144.65 (5)	N1—C3—H3B	108.7
O10^i^—Ca1—O20	143.21 (5)	N1—C3—C4	114.41 (17)
O10—Ca1—N10^i^	88.44 (5)	H3A—C3—H3B	107.6
O10^i^—Ca1—N10^i^	25.30 (4)	C4—C3—H3A	108.7
O10^i^—Ca1—N20	135.44 (5)	C4—C3—H3B	108.7
O10—Ca1—N20	160.78 (5)	N1—C1—H1A	109.1
O1—Ca1—Ca1^i^	78.79 (4)	N1—C1—H1B	109.1
O1—Ca1—O3^i^	156.87 (5)	N1—C1—C2	112.29 (19)
O1—Ca1—O10	81.98 (5)	H1A—C1—H1B	107.9
O1—Ca1—O10^i^	78.98 (5)	C2—C1—H1A	109.1
O1—Ca1—O90	96.27 (5)	C2—C1—H1B	109.1
O1—Ca1—H90A	84.2	N2—C7—H7A	109.0
O1—Ca1—H90B	89.9	N2—C7—H7B	109.0
O1—Ca1—O11^i^	81.03 (5)	N2—C7—C8	113.01 (18)
O1—Ca1—O20	82.91 (5)	H7A—C7—H7B	107.8
O1—Ca1—O21	123.37 (5)	C8—C7—H7A	109.0
O1—Ca1—N10^i^	78.50 (5)	C8—C7—H7B	109.0
O1—Ca1—N20	104.00 (5)	N4—C13—H13A	108.8
O90—Ca1—O10^i^	138.09 (5)	N4—C13—H13B	108.8
O90—Ca1—O10	74.79 (5)	N4—C13—C14	113.91 (18)
O90—Ca1—H90A	17.0	H13A—C13—H13B	107.7
O90—Ca1—H90B	17.1	C14—C13—H13A	108.8
O90—Ca1—O11^i^	170.78 (5)	C14—C13—H13B	108.8
O90—Ca1—O20	75.35 (5)	N2—C5—H5A	108.7
O90—Ca1—O21	98.27 (6)	N2—C5—H5B	108.7
O90—Ca1—N10^i^	163.03 (5)	N2—C5—C6	114.20 (18)
O90—Ca1—N20	86.32 (5)	H5A—C5—H5B	107.6
H90A—Ca1—H90B	28.4	C6—C5—H5A	108.7
O11^i^—Ca1—H90A	154.5	C6—C5—H5B	108.7
O11^i^—Ca1—H90B	168.6	N3—C9—H9A	109.2
O11^i^—Ca1—O20	95.53 (5)	N3—C9—H9B	109.2
O11^i^—Ca1—N10^i^	24.97 (4)	N3—C9—C10	112.26 (18)
O11^i^—Ca1—N20	85.79 (5)	H9A—C9—H9B	107.9
O20—Ca1—H90A	61.9	C10—C9—H9A	109.2
O20—Ca1—H90B	90.2	C10—C9—H9B	109.2
O20—Ca1—N10^i^	119.41 (5)	N4—C15—H15A	108.8
O20—Ca1—N20	24.95 (5)	N4—C15—H15B	108.8
O21—Ca1—O10	154.57 (5)	N4—C15—C16	113.88 (18)
O21—Ca1—O10^i^	119.29 (5)	H15A—C15—H15B	107.7
O21—Ca1—H90A	95.0	C16—C15—H15A	108.8
O21—Ca1—H90B	114.8	C16—C15—H15B	108.8
O21—Ca1—O11^i^	76.23 (6)	C11—C12—H12A	109.5
O21—Ca1—O20	49.41 (5)	C11—C12—H12B	109.5
O21—Ca1—N10^i^	98.06 (5)	C11—C12—H12C	109.5
O21—Ca1—N20	24.46 (5)	H12A—C12—H12B	109.5
N10^i^—Ca1—Ca1^i^	56.69 (3)	H12A—C12—H12C	109.5
N10^i^—Ca1—H90A	162.2	H12B—C12—H12C	109.5
N10^i^—Ca1—H90B	146.0	C3—C4—H4A	109.5
N10^i^—Ca1—N20	110.57 (5)	C3—C4—H4B	109.5
N20—Ca1—Ca1^i^	166.55 (4)	C3—C4—H4C	109.5
N20—Ca1—H90A	77.7	H4A—C4—H4B	109.5
N20—Ca1—H90B	103.2	H4A—C4—H4C	109.5
O3—P2—O2	111.91 (7)	H4B—C4—H4C	109.5
O3—P2—N3	111.00 (8)	C15—C16—H16A	109.5
O3—P2—N4	118.75 (8)	C15—C16—H16B	109.5
O2—P2—N3	105.47 (8)	C15—C16—H16C	109.5
O2—P2—N4	100.92 (8)	H16A—C16—H16B	109.5
N3—P2—N4	107.63 (8)	H16A—C16—H16C	109.5
O2—P1—N2	103.48 (8)	H16B—C16—H16C	109.5
O2—P1—N1	105.17 (8)	C7—C8—H8A	109.5
O1—P1—O2	111.86 (7)	C7—C8—H8B	109.5
O1—P1—N2	116.53 (8)	C7—C8—H8C	109.5
O1—P1—N1	110.04 (8)	H8A—C8—H8B	109.5
N1—P1—N2	109.01 (9)	H8A—C8—H8C	109.5
P2—O3—Ca1^i^	148.41 (8)	H8B—C8—H8C	109.5
Ca1—O10—Ca1^i^	116.69 (5)	C13—C14—H14A	109.5
N10—O10—Ca1	146.30 (11)	C13—C14—H14B	109.5
N10—O10—Ca1^i^	96.30 (10)	C13—C14—H14C	109.5
P1—O2—P2	135.03 (8)	H14A—C14—H14B	109.5
P1—O1—Ca1	169.13 (9)	H14A—C14—H14C	109.5
Ca1—O90—H90A	110.5	H14B—C14—H14C	109.5
Ca1—O90—H90B	110.1	C9—C10—H10A	109.5
H90A—O90—H90B	103.6	C9—C10—H10B	109.5
N10—O11—Ca1^i^	95.74 (10)	C9—C10—H10C	109.5
N20—O20—Ca1	93.84 (11)	H10A—C10—H10B	109.5
N20—O21—Ca1	99.91 (12)	H10A—C10—H10C	109.5
O10—N10—Ca1^i^	58.41 (9)	H10B—C10—H10C	109.5
O11—N10—Ca1^i^	59.30 (9)	C1—C2—H2A	109.5
O11—N10—O10	117.68 (15)	C1—C2—H2B	109.5
O12—N10—Ca1^i^	178.56 (14)	C1—C2—H2C	109.5
O12—N10—O10	120.34 (16)	H2A—C2—H2B	109.5
O12—N10—O11	121.98 (17)	H2A—C2—H2C	109.5
C7—N2—P1	127.28 (13)	H2B—C2—H2C	109.5
C7—N2—C5	116.85 (16)	C5—C6—H6A	109.5
C5—N2—P1	115.81 (14)	C5—C6—H6B	109.5
C11—N3—P2	119.81 (13)	C5—C6—H6C	109.5
C11—N3—C9	116.65 (16)	H6A—C6—H6B	109.5
C9—N3—P2	123.26 (13)	H6A—C6—H6C	109.5
C3—N1—P1	119.79 (13)	H6B—C6—H6C	109.5
C3—N1—C1	117.19 (16)		
			
Ca1—O10—N10—Ca1^i^	168.5 (2)	O2—P1—N1—C3	62.43 (17)
Ca1—O10—N10—O11	170.36 (14)	O2—P1—N1—C1	−100.59 (17)
Ca1^i^—O10—N10—O11	1.87 (17)	O1—P1—O2—P2	42.43 (14)
Ca1^i^—O10—N10—O12	−179.19 (16)	O1—P1—N2—C7	−126.48 (16)
Ca1—O10—N10—O12	−10.7 (3)	O1—P1—N2—C5	56.20 (17)
Ca1^i^—O11—N10—O10	−1.85 (16)	O1—P1—N1—C3	−176.94 (15)
Ca1^i^—O11—N10—O12	179.22 (16)	O1—P1—N1—C1	20.05 (19)
Ca1—O20—N20—O21	−1.6 (2)	N2—P1—O2—P2	−83.81 (13)
Ca1—O20—N20—O22	178.98 (18)	N2—P1—O1—Ca1	9.9 (5)
Ca1—O21—N20—O20	1.7 (2)	N2—P1—N1—C3	−47.99 (18)
Ca1—O21—N20—O22	−178.88 (17)	N2—P1—N1—C1	149.00 (16)
P2—N3—C11—C12	−103.49 (19)	N3—P2—O3—Ca1^i^	122.02 (15)
P2—N3—C9—C10	−114.7 (2)	N3—P2—O2—P1	138.22 (12)
P2—N4—C13—C14	−98.6 (2)	N3—P2—N4—C13	136.32 (16)
P2—N4—C15—C16	111.65 (19)	N3—P2—N4—C15	−60.64 (16)
P1—N2—C7—C8	117.00 (19)	N1—P1—O2—P2	161.86 (12)
P1—N2—C5—C6	116.1 (2)	N1—P1—O1—Ca1	134.7 (4)
P1—N1—C3—C4	−98.4 (2)	N1—P1—N2—C7	108.26 (17)
P1—N1—C1—C2	−127.1 (2)	N1—P1—N2—C5	−69.05 (16)
O3—P2—O2—P1	17.41 (15)	N4—P2—O3—Ca1^i^	−3.46 (19)
O3—P2—N3—C11	172.32 (14)	N4—P2—O2—P1	−109.86 (12)
O3—P2—N3—C9	−1.41 (19)	N4—P2—N3—C11	−56.19 (17)
O3—P2—N4—C13	−96.59 (17)	N4—P2—N3—C9	130.08 (16)
O3—P2—N4—C15	66.46 (16)	C11—N3—C9—C10	71.4 (2)
O2—P2—O3—Ca1^i^	−120.44 (15)	C3—N1—C1—C2	69.5 (3)
O2—P2—N3—C11	50.92 (16)	C1—N1—C3—C4	65.2 (2)
O2—P2—N3—C9	−122.81 (16)	C7—N2—C5—C6	−61.5 (3)
O2—P2—N4—C13	26.05 (17)	C13—N4—C15—C16	−84.1 (2)
O2—P2—N4—C15	−170.90 (13)	C5—N2—C7—C8	−65.7 (2)
O2—P1—O1—Ca1	−108.8 (5)	C9—N3—C11—C12	70.6 (2)
O2—P1—N2—C7	−3.28 (18)	C15—N4—C13—C14	98.7 (2)
O2—P1—N2—C5	179.41 (14)		

Symmetry code: (i) −*x*+1, −*y*+1, −*z*+1.

### Hydrogen-bond geometry (Å, º)

**Table:**

*D*—H···*A*	*D*—H	H···*A*	*D*···*A*	*D*—H···*A*
O90—H90*A*···O20^ii^	0.87	2.21	2.915 (2)	138
O90—H90*B*···O10	0.87	2.58	2.9568 (19)	108
O90—H90*B*···O12	0.87	2.11	2.913 (2)	153
C1—H1*B*···O1	0.99	2.40	2.929 (2)	113
C7—H7*A*···O2	0.99	2.39	2.920 (2)	113
C9—H9*B*···O3	0.99	2.45	2.967 (2)	112

Symmetry code: (ii) −*x*+2, −*y*+1, −*z*+1.

### Selected bond lengths (Å)

**Table:**

Bond	Length (Å)	Bond	Length (Å)
Ca1—O3	2.3324 (13)	P2—O3	1.4722 (13)
Ca1—O1	2.3054 (13)	P1—O1	1.4752 (13)
